# Bis(isoquinolin-2-ium) tetra­chlorido­zincate dihydrate

**DOI:** 10.1107/S1600536814015682

**Published:** 2014-07-23

**Authors:** Elumalai Govindan, Subramani Thirumurugan, Kanniah Rajkumar, Krishnamoorthy Anbalagan, Arunachalam SubbiahPandi

**Affiliations:** a Department of Physics, Presidency College, Chennai 600 005, India; b Department of Chemistry, Pondicherry University, Pondicherry 605 014, India

**Keywords:** crystal structure

## Abstract

In the title compound, (C_9_H_8_N)_2_[ZnCl_4_]·2H_2_O, the tetra­chlorido­zincate ion is located on a twofold rotation axis with the Zn atom on a special position. The crystal packing is stabilized by N—H⋯O and O—H⋯Cl inter­actions.

## Related literature   

For the synthesis of the title compound, see: Anbalagan & Lydia (2011 ▶). For applications of iso­quinoline derivatives, see: Katritsky & Pozharskii (2000 ▶). For a related structure, see: Harrison (2005 ▶). For a description of the Cambridge Crystallographic Database, see: Allen (2002 ▶).
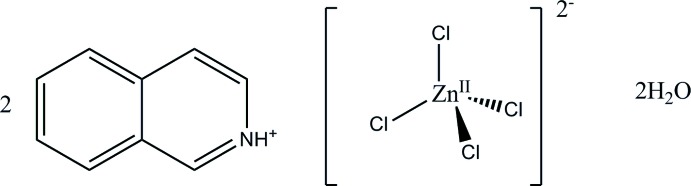


## Experimental   

### 

#### Crystal data  

2(C_9_H_8_N)·Cl_4_Zn·2(H_2_O)
*M* *_r_* = 503.53
Monoclinic, 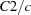

*a* = 11.4337 (5) Å
*b* = 9.9160 (5) Å
*c* = 19.1544 (11) Å
β = 100.120 (6)°
*V* = 2137.87 (19) Å^3^
*Z* = 4
Mo *K*α radiation
μ = 1.66 mm^−1^
*T* = 293 K
0.45 × 0.35 × 0.35 mm

#### Data collection  

Xcalibur, Eos diffractometer
Absorption correction: multi-scan *CrysAlis PRO* (Oxford Diffraction, 2009 ▶) *T*_min_ = 0.502, *T*_max_ = 0.559
5345 measured reflections
1857 independent reflections
1578 reflections with *I* > 2σ(*I*)
*R*_int_ = 0.024
Standard reflections: 0

#### Refinement  

*R*[*F* ^2^ > 2σ(*F* ^2^)] = 0.033
*wR*(*F* ^2^) = 0.088
*S* = 1.04
1857 reflections
129 parameters
4 restraints
H atoms treated by a mixture of independent and constrained refinement
Δρ_max_ = 0.61 e Å^−3^
Δρ_min_ = −0.32 e Å^−3^

### 

Data collection: *CrysAlis CCD* (Oxford Diffraction, 2009 ▶); cell refinement: *CrysAlis RED* (Oxford Diffraction, 2009 ▶); data reduction: *CrysAlis RED*); program(s) used to solve structure: *SHELXS97* (Sheldrick, 2008 ▶); program(s) used to refine structure: *SHELXL97* (Sheldrick, 2008 ▶); molecular graphics: *ORTEP-3 for Windows* (Farrugia, 2012 ▶); software used to prepare material for publication: *SHELXL97* and *PLATON* (Spek, 2009 ▶).

## Supplementary Material

Crystal structure: contains datablock(s) global, I. DOI: 10.1107/S1600536814015682/bt6974sup1.cif

Structure factors: contains datablock(s) I. DOI: 10.1107/S1600536814015682/bt6974Isup2.hkl

CCDC reference: 1012416

Additional supporting information:  crystallographic information; 3D view; checkCIF report

## Figures and Tables

**Table 1:** Hydrogen-bond geometry (Å, °)

*D*—H⋯*A*	*D*—H	H⋯*A*	*D*⋯*A*	*D*—H⋯*A*
N1—H1⋯O1	0.86	1.92	2.747 (4)	161
O1—H1*B*⋯Cl1^i^	0.85 (1)	2.47 (2)	3.270 (3)	157 (4)

Symmetry code: (i) 
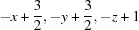
.

**Fig. 1.**
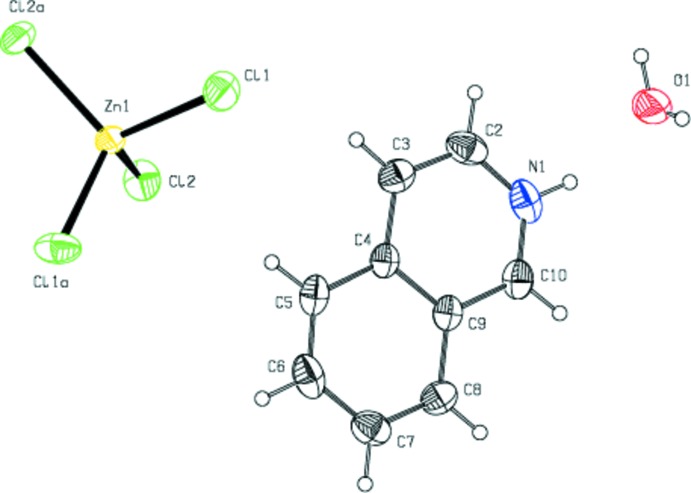
The molecular structure of the title compound, showing the atomic numbering and displacement ellipsoids drawn at 30% probability level.

**Fig. 2.**
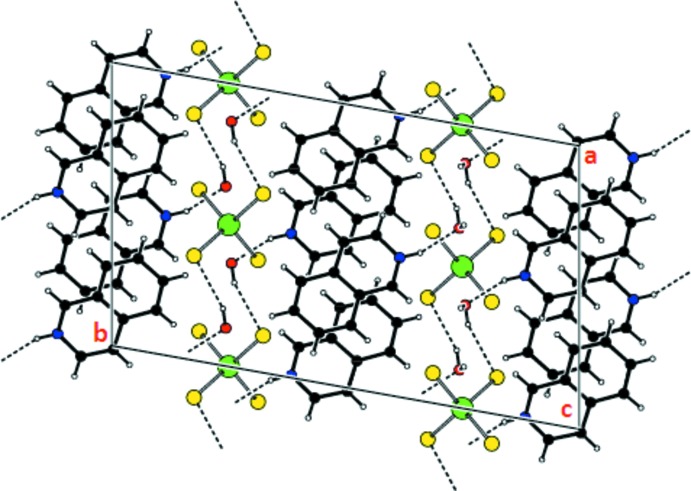
The packing of the molecules viewed down a-axis.

## supplementary crystallographic information

### S1. Comment

Isoquinoline derivatives are of interest in synthesizing new fungicides, insecticides, textile assistants, corrosion inhibitors, dye stabilizers, and pharmaceuticals (Katritsky & Pozharskii, 2000) Against this background and to ascertain the molecular structure and conformation of the title compound, the crystal structure determination has been carried out.

The *ORTEP* plot of the molecule is shown in Fig. 1. The tetrachlorozincate ion is located on a two-fold rotation axis with the Zn atom on the special position. The bond lengths and angles in the title compound are within normal ranges (Allen, 2002) and are comparable with those in related structures (Harrison, 2005).

The crystal packing is stabilized by intermolecular N—H···O and O—H···Cl interactions, which are linking the molecules to a three dimensional network.

### S2. Experimental

Zinc(II) chloride was dissolved in 10 mL(1 mmol) of distilled water. To this isoquinoline in 20 ml of EtOH/HCl mixture (1:5 *v*/*v*) 1 mmol was added in drops. The mixture was heated to 70°C for 2 h and allowed to stand, colorless crystals separated out were filtered and recrystallized using acidified water. X-ray quality crystals were obtained by repeated recrystallization from hot acidified distilled water. Microcrystalline pink color crystal was obtained for analysis.

### S3. Refinement

N and C-bound H atoms were positioned geometrically (N–H =0.84Å, C–H =0.93–0.97 Å) and allowed to ride on their parent atoms, with *U*_iso_(H) =1.5*U*_eq_(C) for methyl H atoms and 1.2*U*_eq_(C,N) for all other H atoms. The coordinates of the H atoms bonded to O were refined with O-H restrained to 0.85 (1)Å and H···H restrained to 1.38 (1)Å.

### Crystal data

**Table:**

2(C_9_H_8_N)·Cl_4_Zn·2(H_2_O)	*F*(000) = 1024
*M**_r_* = 503.53	*D*_x_ = 1.564 Mg m^−^^3^
Monoclinic, *C*2/*c*	Mo *K*α radiation, λ = 0.71073 Å
Hall symbol: -C 2yc	Cell parameters from 1578 reflections
*a* = 11.4337 (5) Å	θ = 3.9–25.0°
*b* = 9.9160 (5) Å	µ = 1.66 mm^−^^1^
*c* = 19.1544 (11) Å	*T* = 293 K
β = 100.120 (6)°	Block, pink
*V* = 2137.87 (19) Å^3^	0.45 × 0.35 × 0.35 mm
*Z* = 4	

### Data collection

**Table:**

Xcalibur, Eos diffractometer	1857 independent reflections
Radiation source: fine-focus sealed tube	1578 reflections with *I* > 2σ(*I*)
Graphite monochromator	*R*_int_ = 0.024
ω scans	θ_max_ = 25.0°, θ_min_ = 3.9°
Absorption correction: multi-scan *CrysAlis PRO* (Oxford Diffraction, 2009)	*h* = −13→13
*T*_min_ = 0.502, *T*_max_ = 0.559	*k* = −11→11
5345 measured reflections	*l* = −15→22

### Refinement

**Table:**

Refinement on *F*^2^	Primary atom site location: structure-invariant direct methods
Least-squares matrix: full	Secondary atom site location: difference Fourier map
*R*[*F*^2^ > 2σ(*F*^2^)] = 0.033	Hydrogen site location: inferred from neighbouring sites
*wR*(*F*^2^) = 0.088	H atoms treated by a mixture of independent and constrained refinement
*S* = 1.04	*w* = 1/[σ^2^(*F*_o_^2^) + (0.0445*P*)^2^ + 1.8258*P*] where *P* = (*F*_o_^2^ + 2*F*_c_^2^)/3
1857 reflections	(Δ/σ)_max_ < 0.001
129 parameters	Δρ_max_ = 0.61 e Å^−^^3^
4 restraints	Δρ_min_ = −0.32 e Å^−^^3^

### Special details

**Table:**

Geometry. All e.s.d.'s (except the e.s.d. in the dihedral angle between two l.s. planes) are estimated using the full covariance matrix. The cell e.s.d.'s are taken into account individually in the estimation of e.s.d.'s in distances, angles and torsion angles; correlations between e.s.d.'s in cell parameters are only used when they are defined by crystal symmetry. An approximate (isotropic) treatment of cell e.s.d.'s is used for estimating e.s.d.'s involving l.s. planes.
Refinement. Refinement of *F*^2^ against ALL reflections. The weighted *R*-factor *wR* and goodness of fit *S* are based on *F*^2^, conventional *R*-factors *R* are based on *F*, with *F* set to zero for negative *F*^2^. The threshold expression of *F*^2^ > σ(*F*^2^) is used only for calculating *R*-factors(gt) *etc*. and is not relevant to the choice of reflections for refinement. *R*-factors based on *F*^2^ are statistically about twice as large as those based on *F*, and *R*- factors based on ALL data will be even larger.

### Fractional atomic coordinates and isotropic or equivalent isotropic displacement parameters (Å^2^)

**Table:**

	*x*	*y*	*z*	*U*_iso_*/*U*_eq_	
C2	0.5564 (3)	0.7834 (3)	0.5659 (2)	0.0638 (9)	
H2	0.6310	0.7446	0.5797	0.077*	
C3	0.5124 (3)	0.8013 (3)	0.49629 (19)	0.0547 (8)	
H3	0.5563	0.7748	0.4621	0.066*	
C4	0.3988 (3)	0.8607 (3)	0.47563 (15)	0.0426 (6)	
C5	0.3464 (3)	0.8850 (3)	0.40473 (16)	0.0551 (8)	
H5	0.3858	0.8605	0.3681	0.066*	
C6	0.2388 (3)	0.9441 (3)	0.39003 (18)	0.0663 (10)	
H6	0.2053	0.9605	0.3430	0.080*	
C7	0.1760 (3)	0.9815 (3)	0.4434 (2)	0.0630 (9)	
H7	0.1018	1.0220	0.4314	0.076*	
C8	0.2223 (3)	0.9590 (3)	0.51179 (18)	0.0543 (8)	
H8	0.1800	0.9832	0.5471	0.065*	
C9	0.3354 (3)	0.8986 (3)	0.52987 (14)	0.0434 (6)	
C10	0.3869 (3)	0.8765 (3)	0.59995 (16)	0.0537 (8)	
H10	0.3462	0.9008	0.6360	0.064*	
Cl1	0.63080 (8)	0.80565 (9)	0.32392 (5)	0.0717 (3)	
N1	0.4918 (3)	0.8221 (2)	0.61579 (15)	0.0630 (8)	
H1	0.5217	0.8100	0.6598	0.076*	
Zn1	0.5000	0.67743 (4)	0.2500	0.04026 (17)	
Cl2	0.39409 (7)	0.54525 (9)	0.31295 (4)	0.0624 (3)	
O1	0.6337 (2)	0.7676 (4)	0.74389 (14)	0.0902 (9)	
H1A	0.647 (3)	0.8552 (12)	0.7531 (15)	0.108*	
H1B	0.7009 (18)	0.737 (3)	0.738 (2)	0.108*	

### Atomic displacement parameters (Å^2^)

**Table:**

	*U*^11^	*U*^22^	*U*^33^	*U*^12^	*U*^13^	*U*^23^
C2	0.0525 (18)	0.0463 (17)	0.086 (3)	0.0002 (15)	−0.0053 (19)	0.0048 (18)
C3	0.0550 (18)	0.0445 (16)	0.067 (2)	−0.0010 (14)	0.0165 (16)	−0.0013 (15)
C4	0.0547 (16)	0.0303 (12)	0.0429 (15)	−0.0075 (12)	0.0092 (13)	−0.0024 (11)
C5	0.079 (2)	0.0473 (16)	0.0403 (16)	−0.0048 (17)	0.0133 (16)	−0.0035 (14)
C6	0.091 (3)	0.0501 (18)	0.0491 (19)	−0.0068 (19)	−0.0103 (19)	0.0034 (15)
C7	0.0583 (19)	0.0479 (18)	0.078 (2)	0.0025 (15)	−0.0020 (18)	0.0034 (17)
C8	0.0575 (18)	0.0441 (16)	0.065 (2)	0.0015 (15)	0.0193 (16)	0.0013 (15)
C9	0.0560 (16)	0.0329 (13)	0.0423 (15)	−0.0078 (13)	0.0112 (13)	−0.0001 (12)
C10	0.073 (2)	0.0450 (15)	0.0430 (16)	−0.0059 (16)	0.0110 (15)	0.0021 (14)
Cl1	0.0560 (5)	0.0770 (6)	0.0835 (6)	−0.0228 (4)	0.0165 (5)	−0.0287 (5)
N1	0.085 (2)	0.0496 (15)	0.0473 (15)	−0.0087 (15)	−0.0087 (15)	0.0038 (12)
Zn1	0.0361 (3)	0.0450 (3)	0.0418 (3)	0.000	0.01239 (19)	0.000
Cl2	0.0637 (5)	0.0713 (5)	0.0561 (5)	−0.0201 (4)	0.0216 (4)	0.0066 (4)
O1	0.0648 (15)	0.149 (3)	0.0565 (15)	0.0270 (18)	0.0102 (13)	0.0027 (17)

### Geometric parameters (Å, º)

**Table:**

C2—C3	1.351 (5)	C8—C9	1.412 (4)
C2—N1	1.362 (5)	C8—H8	0.9300
C2—H2	0.9300	C9—C10	1.385 (4)
C3—C4	1.417 (4)	C10—N1	1.302 (4)
C3—H3	0.9300	C10—H10	0.9300
C4—C5	1.406 (4)	Cl1—Zn1	2.2614 (9)
C4—C9	1.418 (4)	N1—H1	0.8600
C5—C6	1.347 (5)	Zn1—Cl1^i^	2.2614 (9)
C5—H5	0.9300	Zn1—Cl2	2.2697 (7)
C6—C7	1.399 (5)	Zn1—Cl2^i^	2.2697 (7)
C6—H6	0.9300	O1—H1A	0.894 (10)
C7—C8	1.343 (5)	O1—H1B	0.850 (10)
C7—H7	0.9300		
			
C3—C2—N1	120.1 (3)	C7—C8—H8	120.1
C3—C2—H2	119.9	C9—C8—H8	120.1
N1—C2—H2	119.9	C10—C9—C8	121.3 (3)
C2—C3—C4	119.6 (3)	C10—C9—C4	118.9 (3)
C2—C3—H3	120.2	C8—C9—C4	119.8 (3)
C4—C3—H3	120.2	N1—C10—C9	120.6 (3)
C5—C4—C3	123.7 (3)	N1—C10—H10	119.7
C5—C4—C9	118.4 (3)	C9—C10—H10	119.7
C3—C4—C9	117.8 (3)	C10—N1—C2	123.0 (3)
C6—C5—C4	119.7 (3)	C10—N1—H1	118.5
C6—C5—H5	120.2	C2—N1—H1	118.5
C4—C5—H5	120.2	Cl1—Zn1—Cl1^i^	111.58 (6)
C5—C6—C7	122.0 (3)	Cl1—Zn1—Cl2	110.36 (3)
C5—C6—H6	119.0	Cl1^i^—Zn1—Cl2	107.54 (3)
C7—C6—H6	119.0	Cl1—Zn1—Cl2^i^	107.55 (3)
C8—C7—C6	120.3 (3)	Cl1^i^—Zn1—Cl2^i^	110.36 (3)
C8—C7—H7	119.9	Cl2—Zn1—Cl2^i^	109.45 (5)
C6—C7—H7	119.9	H1A—O1—H1B	104.4 (16)
C7—C8—C9	119.8 (3)		

Symmetry code: (i) −*x*+1, *y*, −*z*+1/2.

### Hydrogen-bond geometry (Å, º)

**Table:**

*D*—H···*A*	*D*—H	H···*A*	*D*···*A*	*D*—H···*A*
N1—H1···O1	0.86	1.92	2.747 (4)	161
O1—H1*B*···Cl1^ii^	0.85 (1)	2.47 (2)	3.270 (3)	157 (4)

Symmetry code: (ii) −*x*+3/2, −*y*+3/2, −*z*+1.
